# Institutional modelling: Adding social backbone to agent-based models

**DOI:** 10.1016/j.mex.2022.101801

**Published:** 2022-07-28

**Authors:** Amineh Ghorbani

**Affiliations:** Faculty of Technology, Policy and Management, Delft University of Technology, the Netherlands

**Keywords:** Agent-based modelling and simulation, Institutions, Institutional grammar, Conceptual modelling, Qualitative data, *Computational social simulation*

## Abstract

Institutional modelling is a branch of agent-based modelling and simulation (ABMS) that pays special attention to the social structure by incorporating institutions into these models. Institutions, in this regard, are the rules of the system that shape individual behaviour and interaction. Institutional modelling can make use of the Institutional Grammar (IG), which has 6 pre-specified components to conceptualize institutions. The IG can be used for collecting institutional data for modelling purposes, for coding institutions, and for integrating them into different parts of an agent-based model.

This modelling approach helps capture the structural complexities of social systems in agent-based models. It also supports the explanation of the emergence and dynamics of institutions, not only for better understanding institutions but also for studying social systems, especially for policy analysis.

• Institutional modelling is a branch of agent-based modelling that focuses on and explicitly models the social aspects of socio-ecological-technical systems.
• Institutional modelling supports theory development by enabling modellers to study institutions, and institutional change within, the systems they are embedded in.
• While agent-based modelling is a bottom-up (individual-based) simulation approach, institutional modelling also incorporates top-down institutional structures and aims at studying interactions between bottom-up processes and top-down structural patterns.

## Graphical abstract

**Figure:**
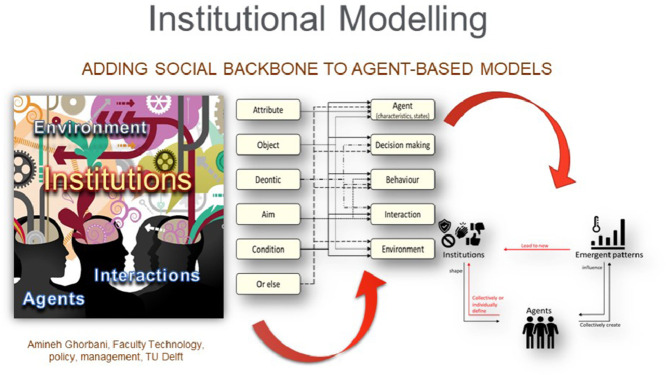

Specifications table

**Table:** 

Subject Area:	Computer Science
More specific subject area:	*Computational Social Science*
Method name:	*Institutional modelling*
Name and reference of original method:	*Agent-based modelling and simulation*
Resource availability:	https://www.comses.net/codebase-release/10eeafa9-f5d4-4534-8109-ffeae0d00b5d/

## *Method details

The method explained in this paper is used to model institutions in simulations of socio-ecological-technical systems. Institutions in this respect are the set of socially shared rules that organize repetitive behaviour in social systems [12] and provide the *social structure* for a system to function [9]. By modelling institutions, these social entities are considered as part of the system, influencing actors while being affected by them.

Institutional modelling builds on agent-based modelling and simulation (ABMS). In ABMS, agents interact with each other and the environment creating patterns of outcomes that are the focus of analysis [2]. In institutional models, institutions are explicitly and formally conceptualized and modelled. Forming the social backbone of the simulation, the agents consider institutions in their decision-making process (e.g., should I drive over the limit?), when interacting with other agents (e.g., should I shake hand?) and with the environment (e.g., what does this signpost imply?).

Since institutions are intangible components in a social system, modelling them is not straightforward: it is unclear where institutions should be included in a model and how. Institutions should not be part of agents because they belong to the society as whole (or part of it). Yet, considering them as the “social environment” does not necessarily indicate how to conceptualize and formulate them. Furthermore, data about institutions is not easy to collect to incorporate into a model given its qualitative nature. Finally, institutions are numerous even within one context and may even be conflicting at times (e.g., drugs are formaly illegal, but a person in a group of friends may feel obliged to take them (norm of the group)). Therefore, incorporating institutions in simulation models is complex and may also need a careful selection process to choose relevant institutional entities that could eventually find their way into the model.

The following sections address all the issues above for better incorporation of institutions into ABMS. The first section introduces a concrete and explicit formalization of institutions. The second section explains the methodological steps for building institutional models, including the data collection process. The final section gives suggestions for selecting institutions to put in models and deciding on the level of detail in which they should be implemented in those models***.***

## Conceptualizing institutions

The institutional modelling method proposed in this paper builds on an existing definition of institutions: “institutions are the rules of the game in a society or, more formally, are the humanly devised constraints that shape human interaction” [12].

The method uses the Institutional Grammar (Crawford and Ostrom [4], extended by Siddiki et al. [14]) to conceptualize institutions. This grammar (also referred to as IG, ADICO or ABDICO) considers institutions as a set of *institutional statements.* Each statement consists of a maximum of 6 components. To better explain each component, consider the following example for an institutional statement: Drivers are forbidden to drive their passenger cars with speeds higher than 130 km/h when on the highway or else they will be fined.

The six components of the ABDICO grammar of institutions are:

1- **[A]ttribute**: the person or group of people to whom the institutional statement applies. In the example above, “drivers” are the attributes of the statement.
2- **[D]eontic type**: the nature of the statement explaining whether it is a “prohibition”, “obligation” or “permission”. In the driving example, “forbidden” shows that the type of institution is “prohibition”.
3- **A[i]m**: the action or outcome associated with the statement. In other words, the action or outcome that the institutional statement applies to. “drive with speeds lower than 130 km/h” is the *aim* component of the driving example.
4- **O[b]ject**: the inanimate or animate part of the statement that is receiver of the action (i.e., aim). “passenger car” is the *object* of the example statement.
5- **[C]ondition**: the situation (i.e., when or where) in which the institution holds. The institution applies to driving “in the highway” in the example above.
6- **[O]r else**: the explicit consequence of not complying with the institution. “Fining” is a typical example of sanctioning.

### Different types of institutions

Not all institutions have all six components of IG. Based on which components a statement has, three different types of institutional statements are proposed [13]. Note that the *object* is an optional component in all types of statements.

1- Rules (A-D-I-C-O): an institutional statement that has these five components (A,D,I,C,O). A rule has an *explicit* and *unique* sanction.
2- Norms (A-D-I-C): an institutional statement that does not have the “or else” component. Non-compliance with norms may have consequences. Yet, there is no explicit or unique sanction attached to a norm. For example, consider the statement: “colleagues shake hands when meeting each other for the first time”. Depending on the context and the people who face this norm, there could be various forms of punishment for not complying with it, such as reconsidering future relations with the non-obedient person or immediate verbal reaction.
3- Shared Strategies (A-I-C): an institutional statement with no sanction (similar to norms) and no deontic attached to it, making it a common routine among people. Following this definition, a person in a given context where the statement applies does not feel any form of legal or social obligation to comply with the strategy. They may or may not decide to follow the common strategy in that context, and there is no implicit nor explicit consequence for not complying. For example, “all students and employees at the university eat between 12 – 12:30”. Eating earlier or later will not have any social or legal consequences if this is a shared strategy. However, the same statement can be considered a norm in a context where eating lunch together is an expectation among a group of employees.

These three different types of statements allow modellers to capture a diverse range of formal and informal institutions in an agent-based model, in a tangible and precise manner.

## Building institutional models

Institutional modelling, similar to other types of modelling approaches, has different phases of 1) system analysis, 2) model conceptualization, 3) detailed design, 4) implementation and 5) evaluation [11].

During the system analysis phase, the modeller studies the system to identify the stakeholders, their decision-making process, the social context and institutions, the stakeholders’ actions and interactions, and the environment they are embedded in. This phase requires extensive data collection (See Section 2.1) that is guided by a modelling question. For example, suppose the modelling question aims to compare various road pricing policies. In that case, the envisioned model is a transport system that includes roads, highways, drivers, tolls, and social and institutional dynamics.

**Fig. 1:**
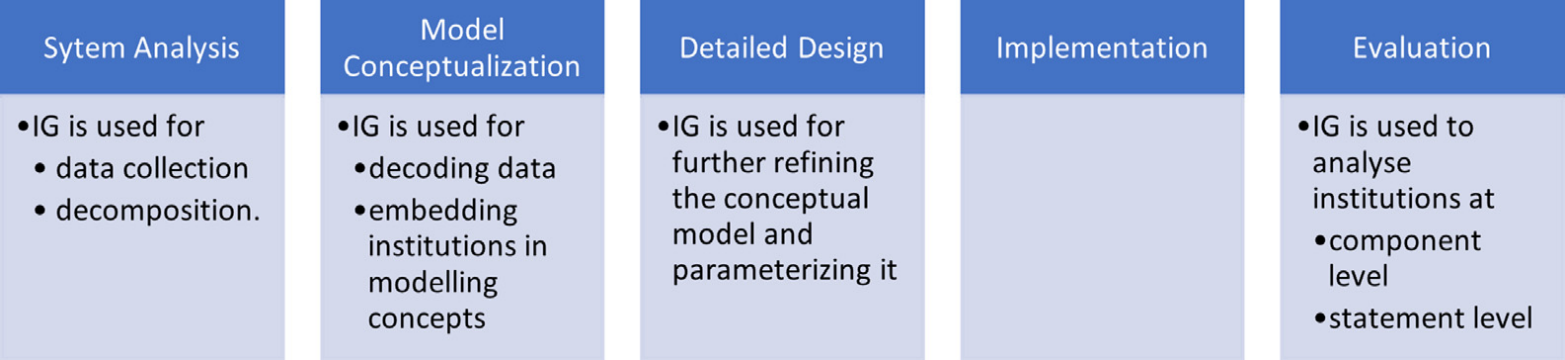
How the IG is used in different phases of the institutional modelling process.

In the conceptualization phase (Section 2.2), the information collected in the previous phase is put together to build the overall concept of the model. This conceptual model can be presented in the form of a narrative or flowchart. More information is added to the conceptual model in the detailed design phase to provide sufficient information to build a software program (e.g., speed limit). Consequently, the detailed design is coded as a simulation, and finally, the simulation is evaluated, experimented with, and analyzed to answer the modelling questions. Figure 1 provides an overview of the institutional modelling process.

### Collecting data for institutional modelling

The identification of institutional statements, parsed according to the IG syntax, involves different methods depending on whether one deals with formal institutions (e.g., public policies) or informal institutions (e.g., spoken or tacitly understood social norms).

### Collecting institutional data from interviews and observations

Identifying informal institutions typically involves interviewing people whose behaviours and interactions are governed by those institutions or observing their behaviours or interactions (ethnographies). Below are steps for employing interviews towards the identification of informal institutions.

1- Select interviewees: Identify the initial set of people to interview in accordance with the agents that are going to be considered in the institutional model. These could potentially be the attributes of the institutions and the agents in the model.
2- Identify the institutions based on the *behaviour* of the interviewees: Identify the main behaviours of the agents in the modelling context(s), and based on those, ask the interviewees whether their behaviour is an individual choice or something that all other agents also do. If the behaviour is shared among the people, it is the” aim” of a potential institutional statement. The animate or inanimate objects are also usually linked to the identified behaviours. The animate objects may represent already identified agents or new ones, thus revealing possible interactions among agents in the model and providing the initial structure of the institutional network.
3- Identify the condition: determine under what state the agents conduct the identified behaviour.
4- Distinguish between norm and shared strategy: For each identified institutional statement in Step 2, ask the interviewee whether they feel social pressure to conduct that behaviour (norm) or routinely do it (shared strategy).
5- Distinguish between norm and rule: For the sake of clarity and simplicity, we assume that all rules are formal and are therefore captured in documents. Therefore, even if a written rule does not have a sanction, we still consider it as a rule if it is extracted from a document. During interviews, if the interviewee is mentioning a rule (with or without a sanctioning), she should be asked for a reference to a certain document where the rule is written. In exceptional cases, the interviewee may be talking about a a social obligation (presence of deontic) and a unique and explicit sanction, in such case, the institution can be considered as a (informal) rule.

Note that more than one person for the same agent type (e.g. households) may need to be interviewed to generalize the data and conclude on the ultimate institutional statements.

### Collection institutional data from existing interviews and field observations

1. Identify potential attributes: Extract each interviewee or other people mentioned in the interview as attributes or animate objects for potential statements.
2. Extract all behaviours associated with the identified attributes in Step 1, and study the data to determine whether they are repeated behaviours among people or individual strategies. A shared behaviour can be considered as an institutional statement.
3. Identify the condition: determine under what condition the agents conduct the identified behaviour.
4. Identify the type of statement: For each identified institutional statement, extract the deontic by identifying whether that behaviour is imposed, permitted, or in case of prohibition, whether the agents are forbidden to conduct that behaviour. If there is no social enforcement nor a sanction-based one, the institution is a shared strategy. If there is some form of social obligation, it is a norm and in exceptational cases if there is a clear and explicit sanctioning mechanism, the statement is an informal rule.

### Collecting institutional data from legal documents

Legal documents contain written and formal rules and regulations that can be extracted using the IG syntax. The challenging issue is that the components of the IG are not always explicitly stated in legal documents, and one sentence may contain several statements combined. Basurto et al. [3] present a 6 step guideline for extracting such information with the IG:

1- Identify and disregard all definitions, titles, preambles, and headings.
2- Distinguish sections and subsections of the document as initial units.
3- For each identified unit in step 2, subdivide the text into sentence-based units as a second level.
4- Use the grammar to decode each sentence-based unit.
5- Identify the type of each decoded unit as rules, norms, or shared strategies. Most of the statement can be considered as rules even if no explicit sanction is mentioned. In some exceptional cases, the document can refer to social obligations (norms) and common routines (shared strategies). Classifying those statements as norms or shared strategies mainly relies on the interpretation of the researcher.
6- If the sentence-based units consist of more than one identified IG statement, subdivide them again into separate statements.

Tables 1 and 2 show an example of a document decoded using the IG. Note that it Table 2, all statements are considered as rules although none of them have explicit sanctions. In [3] however, these statements are assumed to be norms.

**Table 1:** Extracting primary and secondary units of observation in the US Transportation Policy Documents [3].

INITIAL UNITS (SECTION-BASED)	SAMPLE TEXT FROM MPO SETION I34 TEA-2L	SECONDARY UNITS (SENTENCE-BASED)
SUBSECTION	a) 4) Process of development – The process for developing the plans and programs shall provide for consideration of all modes of transportation	Unit 1
	and shall be continuing, cooperative and comprehensive to the degree appropriate, based on the complexity of the transportation problem to be addressed	Unit 2
SUBSECTION	b) Designation of MPO	Unit 3
SUB-SUBSECTION	1) In general – To carry out the transportation planning process required by this section, an MPO shall be designated for each urbanized area with a population of more than 50,000 individuals-	Unit 4
SUB-SUB-SUBSECTION	A) By agreement between the governor and units of general purpose local governor and units of general purpose local government that together represent at least 75% of the affected population (including the central city or cities as defined by the Bureau of the Census): or	Unit 5
SUB-SUB-SUBSECTION	B) In accordance with procedures established by applicable state or local law.	Unit 6
SUB-SUBSECTION	2) Structure – Each policy board of a MPO that serves an area designated as a transportation management area, when designated or redesignated under this subsection, shall consist of-	Unit 7

NOTE: MPO = METROPOLITAN PLANNING ORGANIZATION

**Table 2:** Extracting ADICO statements in the US Transportation Policy Documents [3].

UNIT	SECTION	SYNTAX CODE	DESCRIPTION OF THE ADICO STATEMENT
1	a) 4) process of development	A	MPO (implicit)
		D	Shall - must
		I	Provide for consideration all modes of transportation
		C	[at all times, and in all places, implicit]
		Type	rule
2		A	MPO [implicit]
		D	Shall – must
		I	Be continuing cooperative and comprehensive to the degree appropriate based on the complexity of the transportation problems to be address
		C	[at all times, and in all places, implicit]
		Type	rule
3	b) designation of MPO	discard	Title only
4	b) 1) in general	A	MPO
		D	Shall - must
		I	be designated to carry out the transportation planning process required by this section
		C	for each urbanized area with a population of more than 50,000 individuals-
		Type	rule
5	b) 1) A)	A	MPO [implicit]
		D	Shall – must [implicit]
		I	Be designated to carry out the transportation planning process required by this section [implicit]
		C	Agreement between governor and Govt entities that represent 75% of population
		Type	rule
6	b) 1) B)	A	MPO [implicit]
		D	Shall – must
		I	Be designated to carry out the transportation planning process required by this section [implicit]
		C	In accordance with procedures established by applicable state or local law.
		Type	rule
7	b) 2) structure	A	Each policy board of a MPO that serves an area designated as a transportation management area
		D	Must – shall
		I	Consist of [implicit, in subsections that follow]
		C	When designated or redesgnated under this subsection
		Type	rule

### Embedding institutions in simulation models

This section explains how to incorporate institutions into agent-based models by connecting them to other ABMS components.

Note that institutions, at least at the conceptualization phase should be modelled as independent entities, not as part of agent behaviour, because:

• They are common knowledge recognized by all (or sets of) agents and only sometimes put to practice. An institution, in general, will only change as a result of emerging collective decision(s) or action(s). This change can only be reflected in the model if institutions are considered as explicit entities, at least in the conceptualization phase.
• They are context-specific and should only be available to agents when they are in a specific setting.
• Agents should be able to deliberate about rules, potentially disobey rules, and consequently receive sanctions for non-compliance.
• Institutions may be linked to one another. Therefore, it should be possible to look at institutions independent of agents and at a higher level, similar to the modelling of the physical environment of agents.

**Fig. 2:**
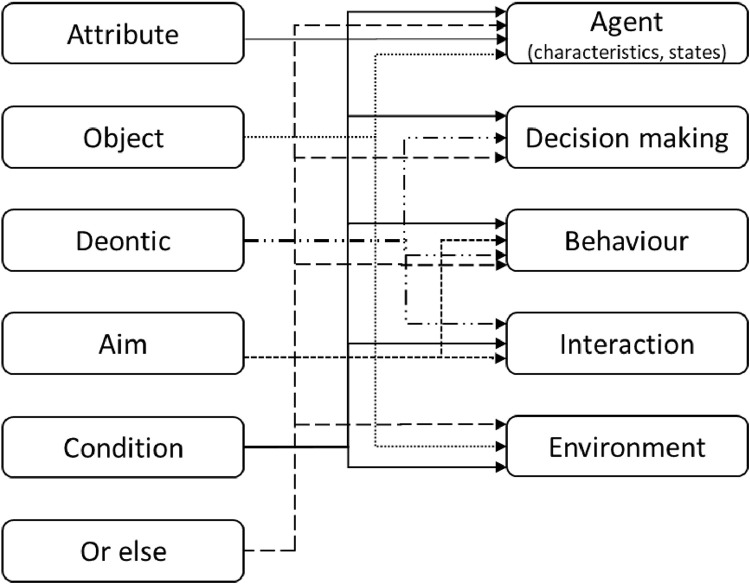
Implementing institutions in agent-based models using the IG.

Modelling institutions implies incorporating the different IG syntactic elements into different parts of an agent-based model. Figure 2 presents how the ABDICO components fit into existing ABMS concepts, if the statements are to be implemented with all details. For a complete agent-based conceptualization that embeds the whole institutional context the reader is referred to [17].

If the attributes of an institution are the agents (one agent, a subset or all) in a simulation, the aim and deontic of the institution directly relate to the actions (i.e., behaviour) or interactions of the agent(s). For example, all drivers (*attribute*/agent) are obliged (*deontic*) to drive below 130 Km/h (*aIm*/ agent action). The deontic, condition and sanction of the institution influence the decision of that agent for conducting that behaviour. This decision can be modelled with any decision making framework such as Theory of planned behaviour or Multi-criteria decision making. The condition part of the IG also allows for connection between the physical and the social contexts. For example, a signpost in an agent's environment indicates the aim (e.g., driving speed) or the sanction (e.g., €50 fine for shoplifting). Or, being on the highway or on a normal street is an environmental state that determines which institution apply in that context. Through the “or else” component, not only the state of the agent can be updated when not conducting the associated behaviour, but the institutions in the simulation can be linked to each other at a higher level.

For example, assume that an agent (driver) wants to decide about her speed and is aware of the institutional rule (signpost). In her decision making, she takes the condition of the rule (in the highway), the deontic (prohibition) and the sanction (100 euros fine) into account. Her decision may be influenced by other factors as well (e.g., being late for work or her age). If she decides to comply, she will conduct the action (drive < 130 km/h). Otherwise, she may have to pay the fine (subject to the monitoring mechanism).

**Fig. 3:**
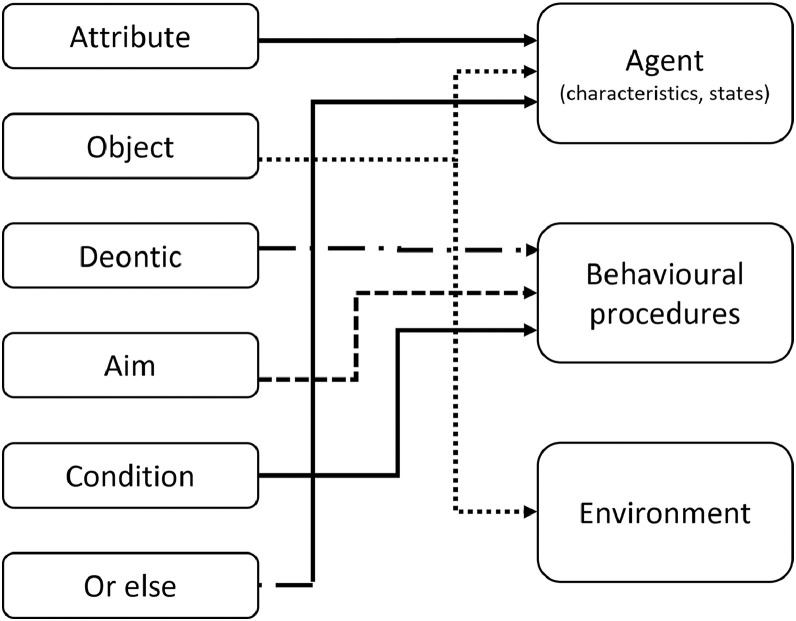
A simplified usage of IG in agent-based models.

Depending on the modelling problem, the conceptualized institutions can be simplified in a model. Figure 3 shows connections between IG statements and agent-based models in a simplified scenario. In other modelling situations, there may be a need for a more complex institutional context and for modelling hierarchies of institutions [5]. In that case, the “or else” component can itself be another institutional statement. For example, for the driving case, the “or else” can be formulated as: “or else the police is permitted to ask for 100 euros fine, if speed > 130 km/h”.

## Final remarks

Having introduced the IG and the way to incorporate institutions into ABMS, the question is, which institutions should we model, and in what detail? The answers to these questions depend on the modelling questions, the modeller's choice, and what they consider relevant to the problem. Below are some guidelines to assist these modelling decisions.

### When to explicitly model institutions

Agent-based models vary significantly in terms of the level of abstraction and detail they capture. Depending on the research question at hand and the availability of data, a model can include complex decision-making behaviour or consist of purely reactive agents. Similarly, a modeller can implement institutions in detail by considering all the IG components (see Figure 2). Or, they can simplify the institutions into conditions that constraint/enable behaviour (see Figure 3). Nonetheless, it is always worthwhile to conceptualize institutions in complete IG statements in the system analysis phase in order to capture and understand the social context before model implementation and to decide about the level of implementation detail later on.

Based on the existing literature and the author's experience in modelling institutions in agent-based models, the following guidelines provide some suggestions on when to implement institutions in social simulations in detail:

1- Policy analysis: Since one of the main usages of ABMS is for policy analysis [2], and given the fact that institutional regulations mostly accompany policy scenarios (e.g., subsidies, taxes), IG-coded institutions enable the modelling of comprehensive policy scenarios (see, for example, [1]).
2- Institutional dynamics: If the goal is to study institutional dynamics, modelling them in detail can facilitate such a study. For example, in [6], for each component of the IG, there is a list of options that the agents can choose from. By putting the selected components together the agents define individual strategies which are similar in structure to shared strategies. The agents collectively define institutions by voting: i.e., they suggest their own individual strategy as the preferred rule. These institutions change over time depending on the satisfaction level of the agents.
3- Collective institutional patterns: When the behaviour of a crowd of people is the subject of study, social norms and rules that guide the overall behavioural patterns play an important role. An explicit representation of these social rules allows the modeller to parameterize these rules, study the effect of these rules on the emergent behaviour of the crowd, and study how the emergent behaviour can influence the general structural rules of the society that lead to new institutions (e.g., [8,5]).

### Static or dynamics institutions

If the goal of the agent-based model is *not* to study institutional dynamics, institutions can primarily be modelled in a static manner. I.e., the five components of the IG can be hard-coded into the simulation to give a pre-defined social structure to the model that influences agent behaviour while the agents do not change the institutions (e.g., [16]). The assumption here is that the time horizon in the model is short enough to consider institutions as unchanged. Nonetheless, even with hard-coded institutions, the modeller can specify various simulation scenarios with different values for the IG components to study different institutional configuration and their influence on the system.

**Fig. 4:**
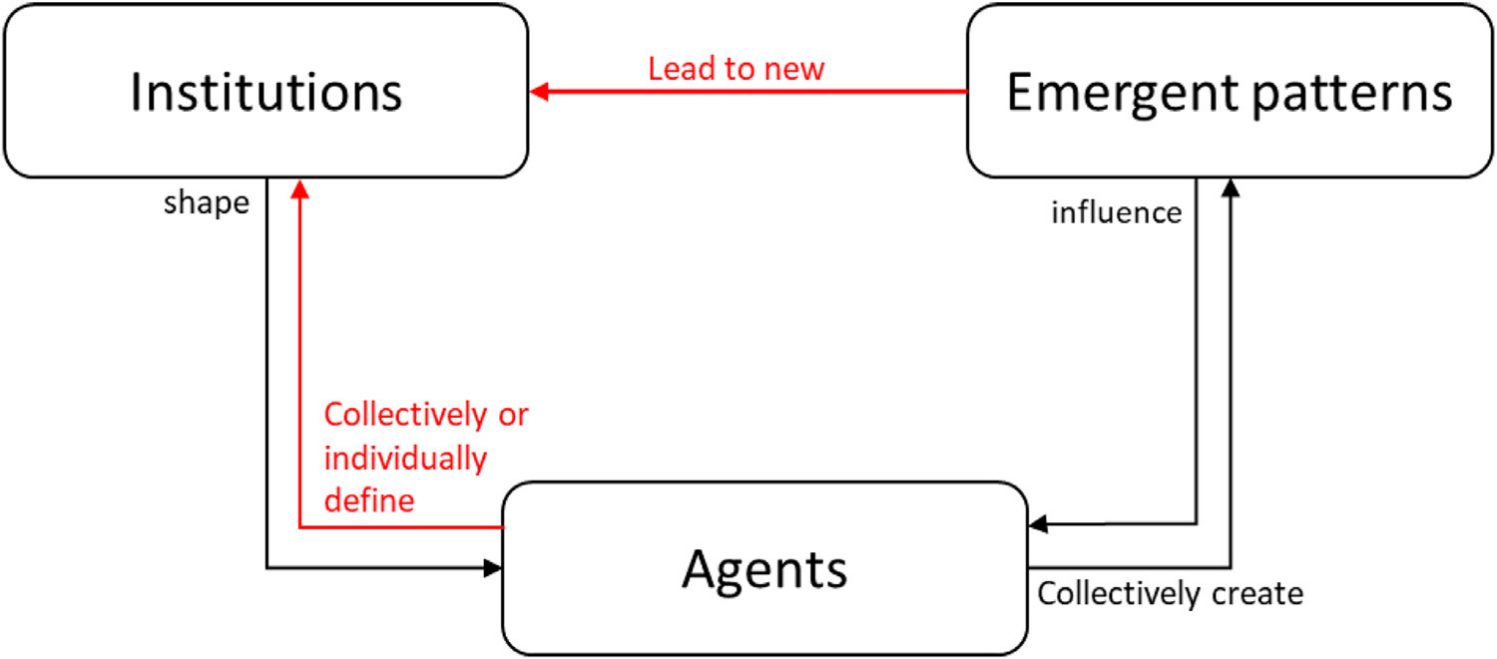
Static vs. Dynamic institutional modelling. The red arrows highlight the added functionalities of dynamic modelling.

The IG also allows for dynamic institutional modelling. It is possible to enable the agents to change the values of the different components of the IG and even introduce new ones by putting the components together (e.g., [6,15]. By allowing agents to use a language to define institutions, these social structures are not pre-defined by the modeller but are emergent from the simulation. Furthermore, given the explicit definition of an institution, it is possible to model agents that can perceive existing or emerging institutions and can consciously attempt to change them. Figure 4 shows the differences between static and dynamic modelling of institutions.

To summarize, some of the possibilities that institutional modelling introduces are:

1- The change of top-down social structures by agents: in simulations, global patterns and structures are extracted from the analysis of data by the modeller as an outcome of the simulation. With institutional modelling, it is possible to allow the agents to perceive and distinguish institutions as intermediate outcomes and possibly attempt to change them individually or through deliberation with other agents during simulation runs (see example: [7]).
2- The evolution of institutions: By gradually introducing the “Deontic” and “Or else” components to institutional statements, it is possible to study the transition of shared strategies to norms, and norms to rules and vice versa. This perspective implies that shared behavioural strategies among agents can eventually take the shape of norms where agents feel the obligation to comply with the institutions. Likewise, norms may turn out to have repeated and similar sanctioning mechanisms in the face of non-compliance and therefore turn into (formal) rules. The reverse of this evolutionary process is also possible: rules become so internalized that they lose the "or else" component and turn into norms, and norms become so regular that they lose their obligatory nature and become shared strategies.
3- The formulation of new institutions by agents: the IG provides a grammar for agents to build institutions in simulations. By providing a list of possibilities for each component of the IG, the agents can create new institutions by combining different options (see [6,15]).

### Networks of institutions

With the IG, it is possible to build a different form of social network where institutions define the relationship between actors [10], mainly though objects as explained earlier. For example, traffic rules define the relationship between a driver and a policewoman (i.e., one permitted to sanction the other). This type of social network can be used in agent-based models and provides a new means to capture interactions among agents and between various institutions in a given context.

## Declaration of Competing Interest

The authors declare that they have no known competing financial interests or personal relationships that could have appeared to influence the work reported in this paper.
